# Rhubarb as a Potential Component of an Anti-Inflammatory Diet

**DOI:** 10.3390/foods14244219

**Published:** 2025-12-09

**Authors:** Joanna Kolodziejczyk-Czepas, Jan Czepas

**Affiliations:** 1 Department of General Biochemistry, Faculty of Biology and Environmental Protection, University of Lodz, 90-236 Lodz, Poland; 2 Department of Oncobiology and Epigenetics, Faculty of Biology and Environmental Protection, University of Lodz, 90-236 Lodz, Poland; jan.czepas@biol.uni.lodz.pl

**Keywords:** *Rheum*, inflammation, civilization diseases, food, phytochemicals, fiber

## Abstract

As a critical element of many civilization diseases, inflammation is a challenge for research on the development of effective treatment or preventive therapies. One of the fundamental approaches in reducing the chronic inflammatory response is to target modifiable risk factors, such as lifestyle and diet. Rhubarb (*Rheum* L.) is one of the oldest medicinal plants, used in the ethnomedicine of different cultures and widely known for its use in Chinese traditional medicine. Rhubarb plants are rich in bioactive phytochemicals, belonging to diverse classes of specialized plant metabolites, which contribute to a broad spectrum of their biological activities, including the alleviation of inflammation of various etiologies. This narrative review focuses on the health-promoting properties of rhubarb as a dietary ingredient, with a particular emphasis on its anti-inflammatory properties as a functional dietary component. Rhubarb is also a rich source of dietary fiber and polysaccharides, which can evoke anti-inflammatory and immunomodulatory effects as well. Different ways of rhubarb processing can significantly affect its chemical composition and biological activity, which may result from the degradation of temperature-sensitive substances such as glycosides. Aglycone release may enhance the bioactive properties of plant materials. Both rhubarb-derived extracts and single compounds can induce various anti-inflammatory effects through numerous mechanisms at the molecular, cellular, and systemic levels. Therefore, rhubarb demonstrates promising antioxidant and anti-inflammatory properties, which may contribute to therapeutic strategies targeting obesity, cardiovascular diseases, and other inflammation-associated disorders. Additionally, it may support the proper functioning of the digestive system.

## 1. Introduction

Inflammation has evolved as a multistep and multilevel process constituting the first line of the animal body’s defense against harmful stimuli such as injuries, pathogens, and other irritants. As a defensive response, inflammation involves a sequence of diverse processes, including initiation of the inflammatory response (mediated by activation of the hemostatic and the immune systems) and its controlled development, as well as suppression and recovery. However, both the development and physiological outcome of acute and chronic inflammatory responses differ substantially. While acute inflammation is believed to be crucial for healing and recovery, chronic inflammation may become a silent promoter of many health problems, including civilization diseases [1]. The etiology of not only physical but also mental disorders that dominate current morbidity and mortality worldwide is associated with chronic inflammation. According to the literature, more than 50% of all deaths in Western civilization are related to inflammation-associated diseases such as cardiovascular diseases, diabetes mellitus, cancer, non-alcoholic fatty liver disease (NAFLD), autoimmune diseases, and chronic kidney disease, as well as neurodegenerative disorders. Moreover, social, environmental, and lifestyle factors can promote chronic inflammation, enhancing its harmful effects at the systemic level [2]. It has been well-evidenced that certain non-modifiable and modifiable factors, such as age, smoking, unhealthy diet, sedentary lifestyle, obesity, hormones, stress, and irregular sleep patterns, promote low-grade chronic inflammation [2,3].

A growing body of data has indicated a link between a diet rich in anti-inflammatory ingredients, a reduction in levels of inflammation markers, and disease outcomes in various adult populations. Key elements of a healthy diet comprise various plant-derived compounds, including vitamins, ω-3 fatty acids, polyphenols, dietary fiber, and prebiotics [4,5,6,7]. The concept of an anti-inflammatory diet includes targeting different aspects of human physiology, both at the cellular, tissue, and systemic levels. The anti-inflammatory effects of various dietary components are attributed to their ability to modulate pro-inflammatory gene expression and block the initiation of the pro-inflammatory signaling pathways, as well as suppress inflammation-associated oxidative stress. At the systemic level, natural anti-inflammatory substances may support the gut microbiota, regulate the adipose tissue metabolism, or enhance physiological antioxidant and detoxification mechanisms [8,9,10,11] (Table 1).

Natural substances, which can display anti-inflammatory activity, belong, i.a., to low-molecular-weight specialized plant metabolites, e.g., polyphenol compounds, and can be found in many plants. Among others, the species belonging to the *Rheum* L. genus (rhubarb plants) are known to be rich in many health-beneficial bioactive compounds, including those of anti-inflammatory and/or immunomodulatory activities. The *Rheum* L. genus (Polygonaceae) comprises approximately 60 species of perennial herbs and is divided into seven sections, i.e., *Acuminata*, *Deserticola*, *Rhapontica*, *Palmata*, *Spiciforma*, *Globulosa*, and *Nobilia* [12]. Rhubarbs are naturally present and/or cultivated in different regions of the world and have been used for centuries as vegetables for culinary and as herbs for medicinal purposes in various cultures. Their use is highly organ-specific. Both aerial and underground parts of rhubarbs have been used, but as food components, the aerial parts (petioles) are used, and for medicinal use, mostly the underground parts (roots and rhizomes) are used. The leaves are toxic due to their high oxalic acid content. Bioactive phytochemicals displaying anti-inflammatory activity of the *Rheum* plants include mainly stilbene, flavonoid, and some anthraquinone compounds; also, other substances synthesized by rhubarbs, such as fiber and other polysaccharides, may exhibit immunomodulatory and anti-inflammatory actions resulting in direct and indirect beneficial effects on human health [13]. Curative actions of various rhubarb-derived preparations known for centuries to treat different ailments have been elucidated in detail, owing to the good modern characterization of the phytochemical composition of many rhubarb species. The beneficial health properties of rhubarbs have been attributed to specific extracts and to isolated compounds belonging to several principal classes of specialized plant metabolites. Although much research has already been performed, many rhubarb-specific properties have to be further elaborated in more detail, including their anti-inflammatory and immunomodulatory actions, especially due to the involvement of chronic inflammation in the pathogenesis of a number of diseases.

Therefore, this work is focused on the health benefits of different rhubarb (*Rheum* L.) species, related to their ability to mitigate inflammation of various etiologies. To provide a broader perspective on the anti-inflammatory potential of these plants, we present both the direct and indirect anti-inflammatory effects of rhubarb-derived products and extracts, particularly in the context of the pathophysiology of lifestyle-related diseases. Given the extensive body of literature concerning individual phytochemicals that are present both in rhubarbs and other plants, this review is based primarily on studies specifically addressing rhubarb-derived substances and extracts. Where relevant, references to the biological activity of specific compounds have been included to guide the reader through the scientific background.

Our review is based on data published up to October 2025, originating from journals indexed in international databases, including Medline/PubMed, Scopus, ScienceDirect (Elsevier), and SpringerLink (ICM). The main search criteria included a combination of the “Rheum” or “rhubarb” words with “anti-inflammatory”, “inflammation”, “civilization diseases”, “lifestyle diseases”, “antioxidant”, oxidative stress”, “metabolic disorders”, “diet”, “fiber”, and “fibre”. While searching for ethnomedicinal data, local scientific publications not indexed in these databases were also taken into consideration.

## 2. Rhubarbs as Medicinal Plants

With over two thousand years of ethnomedicinal use (including in Traditional Chinese Medicine, TCM), various rhubarb species belong to the oldest known medicinal plants [14]. The medicinal properties of rhubarb are primarily attributed to its roots and rhizomes. However, the use of other plant parts, such as petioles (also known as stalks) and leaves, in traditional medicine is well-documented (Table 2).

## 3. Phytochemical Profile of Rhubarb and Rhubarb Processing

**Figure 1:**
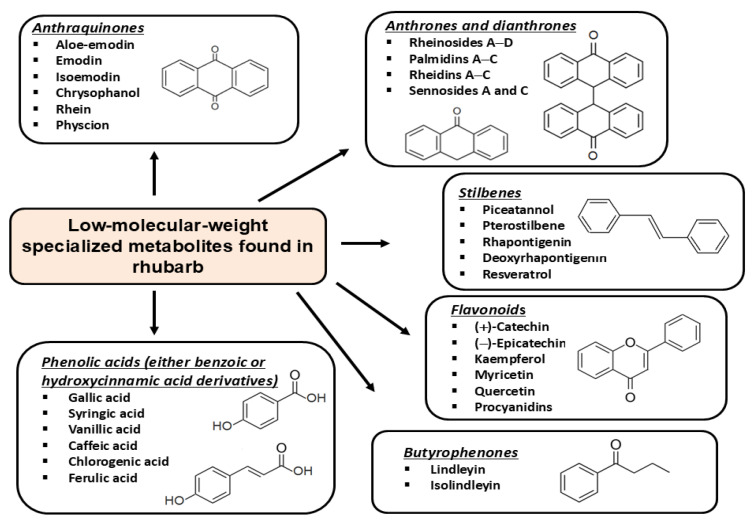
Main groups of the low-molecular-weight bioactive phytochemicals synthesized in rhubarb.

The biological and therapeutic properties of rhubarb extracts result from their diverse phytochemical composition rich in bioactive compounds from various classes of specialized plant metabolites [31]. The phytochemical composition of different rhubarb species and the organ-specific distribution of metabolites have been comprehensively described elsewhere [25,32,33,34]. Briefly, the phytochemical profile of rhubarb includes several main groups of low-molecular-weight specialized metabolites such as anthraquinones, anthrones and dianthrones, stilbenes, butyrophenones and chromones, phenolic acids, and various flavonoid compounds (Figure 1).

However, both the total content of phytochemicals and the composition of phytochemical profile may vary considerably among species [35], and even among cultivars of the same species. A work devoted to a detailed analysis of the influence of various factors on the synthesis and accumulation of 14 active pharmaceutical ingredients in rhubarbs has revealed, e.g., that *R. palmatum* L. was a more effective source of 9 out of 14 of the investigated ingredients when compared with *R. tanguticum* Maxim. ex Balf. [35]. Other study has shown, for instance, that the total phenolics in twenty nine rhubarb varieties ranged from 673 to 4173 mg (of gallic acid equivalents/100 g of dry weight (d.w.)). Moreover, the total anthocyanin content in the aforementioned varieties ranged from 19.8 to 341.1 mg/100 g d.w. [36]. Another study, based on comparative analyses of the phytochemical profile of two rhubarb varieties, i.e., *Red Malinowy* and *Viktoria*, found the most marked differences were in the total flavon-3-ols and anthocyanins, regardless of the season (spring or autumn) of the plant material collection. In *Red Malinowy* samples, the flavan-3-ol content was 195.98 mg/100 g d.w. in spring and 166.93 mg/100 g d.w. in autumn, while its total anthocyanin contents were 96.20 and 81.36 mg/100 g d.w., respectively. In contrast, the plant material of the *Viktoria* variety contained 87.06 and 86.57 mg/100 g d.w. of flavan-3-ols, for spring- and autumn-collected material, respectively. The total anthocyanin contents for the *Viktoria* variety were 4.73 and 4.33 mg/100 g d.w., respectively [37].

Also, the cultivation method of rhubarb significantly influences the concentration of bioactive metabolites, as demonstrated in studies involving chemical, organic, and biological fertilization modes. The chemical fertilization was shown to provide the highest yield (59.16 t × ha^−1^), whereas the unfertilized control yielded 47.27 t × ha^−1^. However, the management based on biological or organic fertilizers resulted in a higher total phenolic content and enhanced antioxidant activity in the harvested plant material, compared to both the unfertilized control and rhubarb treated with chemical fertilization. The biological fertilization was found to provide plant material with the highest antioxidant activity (877.07 mmol Trolox × g^−1^ d.w.), which was more than two times higher than the antioxidant efficacy of the unfertilized rhubarb samples (i.e., 313.17 mmol Trolox × g^−1^ d.w.) [38].

Furthermore, the content and proportions of the main phytochemical compounds can be influenced by various environmental factors, mainly those that depend on the region of plant cultivation, i.e., the level of nutrients in the soil [35], temperature, the level of precipitation, the occurrence of drought(s), the length of the vegetation season, and the altitude of the natural site or cultivation area [39].

One of the main rhubarb cultivation areas is China, where three species, i.e., *R. officinale* (Chinese rhubarb), *R. palmatum*, and *R. tanguticum*, are the most significant. Environmental factors that have been shown to have a significant impact on the levels of active rhubarb pharmaceutical ingredients were as follows: geographic location of the major cultivation areas in China, which determined the soil nutrient level; increasing latitude, which, inside China, negatively influenced the accumulation of all major pharmaceutical ingredients; and a longitude along with the level of rainfall, which influenced the production of anthraquinone compounds. Since, in China, precipitation is lower in regions of lower longitude, such areas should be used for rhubarb cultivation to obtain plants with increased anthraquinone content, if needed for medicinal use [35].

The influence of geographic location on plant morphology, yield, and phytochemical composition has been investigated in a more recent study on *R. tanguticum* material harvested from sites located in the Qinghai–Tibet Plateau [39]. Plant morphology (e.g., plant height, petiole length, stem and root diameter, and root fresh or dry weight) and yield had better characteristics for plants grown for at least 3 years, with the best characteristics for those grown for 4–5 years. Morphological indices and yield were much better for *R. tanguticum* cultivated at lower altitudes. Higher altitude caused lower content of anthraquinone and sennoside compounds; however, total tannin content was increased. High anthraquinone levels were detected in *R. tanguticum* from the Qinghai–Tibet Plateau at sites of the lowest latitude characterized by low levels of precipitation and a slightly basic soil pH. The site of the lowest altitude (2016 m above sea level, m.a.s.l.) induced high levels of lipids and phenolic acids, whereas the site of the highest altitude (3763 m.a.s.l.) induced altitude-associated stress, causing an increase in production of some flavonoids, i.e., luteolin, isovitexin, vitexin, and myricetin, and a decrease in the level of luteolin-7-O-glucuronide. Therefore, *R. tanguticum* plants from high-altitude areas with lower anthraquinone contents have weaker laxative properties, while higher contents of flavonoids increase antioxidant quality of such material [39].

Studies on dependencies between environmental factors, rhubarb cultivation, plant yield, and phytochemical content are of growing importance due to a need to control plant material quality harvested both for food and medicinal purposes. Other important factors are the urgent need to define the best areas for reasonable economic cultivation and, due to overexploitation of wild resources, for conservation sites, especially for rare and endemic species. Another rising problem is climate change, which can substantially alter the possibility of plant cultivation in many areas and pose a threat to endangered species. A recent work on the possible scenarios of climate change on the sites where endemic medicinal Himalayan rhubarb *R. australe* is present either in the wild or as a cultivated plant has shown that predicted climate change may negatively influence areas suitable for this species in the Nepal Himalaya. These areas are expected to decrease in all predicted climate change scenarios in all periods, especially in the years 2081–2100. Northwestern districts of Nepal are predicted to have the greatest losses of suitable habitats, but some districts at altitudes between 3300 and 4400 m.a.s.l. could gain habitats. These new habitats could be sites of possible cultivation or species preservation areas [40]. However, changes in sites of habitats and, particularly, their altitude, could influence the phytochemical profile of plants cultivated in the future, but it remains unclear to what extent.

From the nutraceutical and pharmacological point of view, two sections of the *Rheum* genus, i.e., the *Rhapontica* and the *Palmata*, are of main interest. The *Palmata* section includes anthraquinone-rich species, e.g., *R. officinale* and *R. palmatum*, typically used as medicinal plants. Their roots are available at the pharmaceutical market either as *Rhei radix* or as ingredients of diverse herbal preparations to alleviate gastrointestinal disorders and to treat constipation [41,42]. These rhubarb species are sometimes grown in gardens as ornamental plants as well. In Western countries, the leaf petioles of rhubarb species belonging to the *Palmata* section are seldom used as food. However, they are an ingredient in Asian cuisine. It is noteworthy that petioles of *R. officinale* and *R. tanguticum* have been recently reported to possess significant nutritional value as well [43]. Similar data are derived from studies on the nutritional value of *R. palmatum*. Due to high flavonoid content and good nutritional composition, the stems of this plant have also been proposed as a potential healthy food or dietary supplement [44].

In contrast to members of the *Palmata* section, rhubarbs belonging to the *Rhapontica* section, characterized by a significant content of stilbenes, have been widely used both for medicinal and culinary purposes. Petioles of *R. rhaponticum* (rhapontic rhubarb, Siberian rhubarb), *R. rhabarbarum* (garden rhubarb), and their hybrids, along with dozens of their varieties, are well-known ingredients of the human diet. Furthermore, underground parts of *R. rhaponticum* are key ingredients for preparations exerting estrogenic activity, which is attributed to the abundance of different hydroxystilbenes and their derivatives, such as rhaponticin, deoxyrhaponticin, rhapontigenin, deoxyrhapontigenin, resveratrol, and piceatannol. The *R. rhaponticum* rhizome-containing herbal preparations are administered as an alternative to the conventional hormone replacement therapy, to reduce menopausal vasomotor symptoms, e.g., hot flushes and night sweats. The most well-known preparation based on the *R. rhaponticum* rhizome is a special extract, ERr 731^®^, a registered product with confirmed estrogenic activity [45,46]. Furthermore, extracts of this plant rhizome are also ingredients of many dietary supplements.

Notably, different methods of rhubarb processing can substantially affect the chemical composition of its extracts and modulate their biological activity. Typically, the herbal material (the rhubarb root/rhizome, mainly) intended for medicinal purposes is cleaned, lacked impurities, sliced, and dried. The Chinese Pharmacopeia also provides other traditional medicine-based ways of rhubarb processing, through employing thermal treatment. Cooked rhubarb is obtained by stewing or steaming the raw plant material with wine until it becomes uniformly black, and the rhubarb charcoal is produced by stir-frying rhubarb to obtain a visibly charred surface and a burnt brown interior [41]. Thermally processed rhubarb tends to cause fewer side effects than raw rhubarb and rarely leads to diarrhea [47].

The edible petioles of different rhubarb species are usually thermally processed (by boiling, stewing, or baking). Thermal processing may alter the levels and profiles of bioactive compounds. These changes may result from the release of substances from plant tissues or from the degradation of temperature-sensitive compounds, such as glycosides. Recent studies on the scorch processing (i.e., stir-baking at 180 °C) of raw rhubarb (*R. tanguticum*) demonstrated that this type of rhubarb processing led to the pyrolysis of anthraquinone glucosides and the generation of their aglycones, exerting higher biological activity. Rhubarb samples baked at 180 °C for 50 min had the optimal degree of scorching. Anti-inflammatory properties of the unprocessed and scorched rhubarb were studied in an animal model of thromboinflammation, a complex pathological condition characterized by an inflammatory response evoked by the hyperactivation of the blood coagulation cascade and thrombus formation. Comparative analyses of the biological activity of the unprocessed and scorched rhubarb revealed that this type of thermal processing enhanced its biological activity and anti-thromboinflammatory efficacy. Furthermore, the scorched rhubarb did not exert purgative side effects [48]. Other work demonstrated that both fast and slow stewing, as well as baking of *R. rhaponticum* petioles, increased the total polyphenolic compounds and antioxidant capacity when compared to raw petioles. This enhancement was attributed to the heat-induced degradation of plant tissues, facilitating the release of bioactive substances [49]. In addition, rhubarb processing can affect its oxalate content, particularly when it is cooked with substances that bind or absorb oxalates [50,51]. It has been suggested that cooking rhubarb petioles with milk may reduce the intake of soluble oxalates, which is especially beneficial for individuals prone to the formation of kidney stones. Experimental in vitro simulations of raw and cooked rhubarb (*R. rhabarbarum*) digestion and oxalate bioaccessibility revealed that the soluble oxalate level in a typical serving of boiled rhubarb may be reduced even by 70.3% when cooked in the presence of milk. The reduction in oxalate levels may result from the presence of calcium in the cooking medium. The content of soluble oxalates from rhubarb products can also be reduced by trapping them within dietary fiber [52].

The nutritional value of rhubarb-containing food may be enhanced by combining it with other foods, especially those with a significant health-promoting potential. Based on available data, it seems that one of the most promising actions could be enhancing the antioxidant capacity of rhubarb preparations with other foods rich in antioxidants, such as anthocyanins and flavonoids. A recent work by Korus and Korus [53] has provided an interesting concept of mixing the *R. rhaponticum* petioles with fruit and vegetable pomace. The rhubarb-based products were sweetened with isomaltulose and enriched with fruit (apple, chokeberry, or black currant) and vegetable (beetroot or carrot) pomace. The rhubarb–pomace combinations contained an increased level of polyphenolic compounds and displayed considerable radical-scavenging activities, established in 2,2′-azino-bis(3-ethylbenzothiazoline-6-sulfonic) acid (ABTS) cation radical- and 2,2-diphenyl-1-picrylhydrazyl (DPPH) radical-based tests. Particularly beneficial was the combination of rhubarb with pomace derived from fruits with the highest total content of polyphenols, including anthocyanins, such as chokeberry and blackcurrant. Compared to rhubarb samples without pomace, the addition of chokeberry and blackcurrant pomace enhanced the ABTS^•+^-scavenging activity by 374% and 321%, respectively. The DPPH^•^-scavenging activity was enhanced by 317% and 249%, respectively.

Moreover, the literature indicates that rhubarb stalk juice may be used as an additive during food processing to stabilize its polyphenolic components. The rhubarb juice has been shown to effectively inhibit browning in fresh-cut fruits and vegetables [54]. It also reduced undesirable color changes in processed products such as strawberry jams [55] and apple purées [56]. In studies on improving the properties of strawberry jams, adding the rhubarb juice enhanced their ABTS^•+^- and DPPH^•^-scavenging abilities, along with enhancing the reducing power [55]. Recently, rhubarb stalk powder has also been investigated for its potential as a functional ingredient in bread. Increasing the proportion of rhubarb powder led to a rise in fiber, ash, and fat content, as well as enhanced antioxidant activity and the total phenolic content of the product. On the other hand, an increase in the content of rhubarb powder in the composition of bread was associated with a weakening of its sensory parameters. Bread samples containing 4% of rhubarb powder were established to have the highest sensory scores, including odor, flavor, and overall impression [57].

## 4. Rhubarbs as a Source of Dietary Fiber

**Figure 2:**
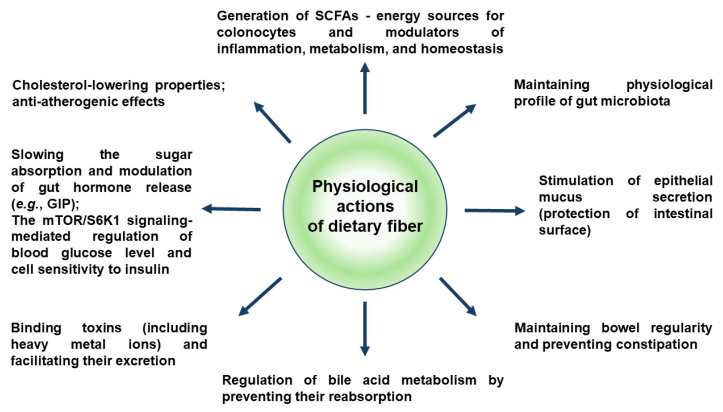
Physiological functions of dietary fiber, including anti-inflammatory effects. Abbreviations: GIP—glucose-dependent insulinotropic peptide; mTOR/S6K1 pathway—the mammalian target of rapamycin/p70 ribosomal protein kinase 1 signaling pathway; SCFAs—short-chain fatty acids.

In addition to products strictly dedicated to alleviating menopausal complaints and concoctions for gastrointestinal tract disorders, the contemporary food and pharmaceutical market offers numerous dietary supplements and other products containing rhubarb petioles or extracted fiber. Although the fiber may not be widely recognized as a typical anti-inflammatory agent, its direct and indirect beneficial effects on overall human health (Figure 2), its key role in maintaining the gastrointestinal system functionality, and its ability to mitigate inflammatory processes (including diet- and lifestyle-induced low-grade inflammation) are well-documented [58].

Several clinical studies revealed that high fiber intake improves inflammatory markers in obesity and/or the metabolic syndrome-diagnosed patients [59]. As a source of short-chain fatty acids (SCFAs) such as acetate, propionate, and butyrate, fermentable dietary fiber is an active player in maintaining systemic homeostasis, including the modulation of metabolism, immunity, and inflammatory response. The SCFAs display an affinity to the G-protein-coupled receptors (GPCRs), including the FFAR2/GPR 43, GPR 41, and GPR109A, and, therefore, the SCFAs influence the response of monocytes/macrophages, dendritic cells, colonic epithelial cells, intraepithelial lymphocytes, and adipose tissue [60]. The SCFAs have also been found to target the olfactory G protein-coupled receptor (Olfr-78/OR51E2), which is a regulator of glucose homeostasis [61] and may be involved in the modulation of intestinal inflammation [62].

In general, dietary fiber is classified into two categories, i.e., soluble dietary fiber (SDF; containing pectin and non-starch polysaccharides) and insoluble dietary fiber (IDF; consisting of cellulose, hemicellulose, and lignin). Raw and cooked petioles of culinary rhubarbs belonging to the *Rhapontica* section contain various types of dietary fiber and may be competitive with other fiber-rich food products. For instance, 140 g of stewed rhubarb was found to contain 3.8 g of the total NSPs (non-starch polysaccharides), 2.2 g of insoluble NCPs (non-cellulosic polysaccharides), and 0.7 g of soluble NCP fiber [63]. The total dietary fiber content in fresh and frozen rhubarb petioles was found to be 741 g/kg d.w. This included 659 g/kg d.w. of IDF and 82 g/kg d.w. of SDF [64]. Moreover, according to comparative studies on the fiber content in different vegetables and fruits, its level in rhubarb petioles was surprisingly high. The petioles contained 26.7% of insoluble fiber, whereas 54.9% were residues after digestion with Driselase. For a comparison, insoluble fiber content in apples was 9.8%, and the digestion residue content amounted 46.2%. For carrots, the above fiber contents were of 10.3% and 34.6%, respectively [65].

The nutritional value and fiber content in leaf petioles of *R. officinale* and *R. tanguticum* have also been revealed [43]. Interestingly, dried petioles of both plants contained significant amounts of fiber (11.71% and 13.17%, respectively) and essential polyunsaturated fatty acids (2.24 g/100 g and 2.84 g/100 g, respectively). In addition, *R. officinale* and *R. tanguticum* were abundant in minerals (4.27 g/100 g and 5.74 g/100 g of samples, respectively). The dominating mineral component of rhubarb petioles from both species was calcium (3.28 g/100 g and 4.28 g/100, respectively), exceeding its recommended daily intake of 1–1.30 g/day [43].

## 5. Diverse Mechanisms of Anti-Inflammatory Action of Rhubarb-Derived Extracts and Foods

Phytochemicals exert anti-inflammatory properties by targeting distinct molecular and cellular levels of pro-inflammatory response, including signaling pathways mediated by MAPKs and other kinases, transcription factors (e.g., NF-κB), production of inflammatory mediators, activity of pro-inflammatory enzymes, and even the modulation of cell differentiation and antioxidant defense. Their different anti-inflammatory activities result from diverse structures and bioavailability. Among the main groups of rhubarb metabolites, the anti-inflammatory activity was primarily found for stilbenes, flavonoids, and some anthraquinones.

### 5.1. Suppression of Pro-Inflammatory Response at Different Molecular and Cellular Levels

**Figure 3:**
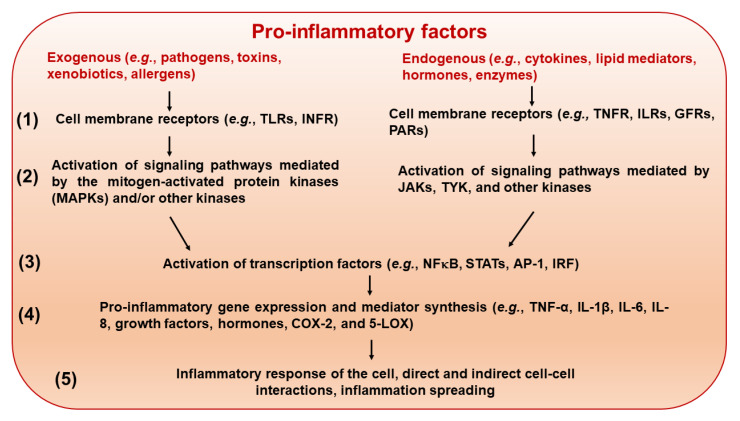
Molecular levels and main mediators of inflammatory response. (1) External and endogenous stimuli initiate the inflammatory response by activating various cell membrane receptors, including pattern recognition receptors and cytokine receptors. (2) Depending on the receptor involved, distinct signaling pathways are triggered and mediated by kinases. (3) The pro-inflammatory signaling targets the nucleus and modulates the activity of transcription factors. (4) Gene expression of pro-inflammatory mediators is triggered. (5) The cellular inflammatory response is developed and propagated to surrounding cells and tissues. Abbreviations: 5-LOX—5-lipoxygenase; AP-1—activating protein-1; COX-2—cyclooxygenase 2; GFRs—growth factor receptors; IL-1β, -6, and -8—interleukins 1β, -6, and -8; ILRs—interleukin receptors; INFR—interferon receptor; IRF—interferon regulatory factor; JAKs—Janus-activated kinases; MAPKs—mitogen-activated protein kinases; NF-κB—nuclear factor kappa-light-chain-enhancer of activated B cells; PARs—protease-activated receptors; STATs—signal transducer and activator of transcription factors; TLRs—Toll-like receptors; TNF-α—tumor necrosis factor α; TNFR—tumor necrosis factor receptor; TYK—tyrosine-protein kinase.

Due to a multilevel character and complexity of the inflammatory response [66,67,68] (Figure 3), its suppression may occur through regulation of various mechanisms at the molecular, cellular, and systemic levels, including regulation of transcription factors responsible for expression of genes encoding inflammatory mediators, inhibition of pro-inflammatory enzymes (e.g., by non-steroidal anti-inflammatory drugs; NSAIDs), and targeting receptors using steroidal drugs.

The most common anti-inflammatory approach is inhibiting pro-inflammatory enzymes involved in arachidonic acid metabolism, specifically cyclooxygenase-2 (COX-2) and 5-lipoxygenase (5-LOX). The COX-2 isoenzyme is responsible for the generation of pro-inflammatory prostanoids. 5-LOX promotes inflammation by catalyzing the biosynthesis of leukotrienes (LTs), a distinct group of the arachidonate-derived mediators of inflammation. The widespread use of NSAIDs (such as acetylsalicylic acid, ibuprofen, and naproxen) as COX-2 inhibitors, is a consequence of their availability without a prescription and a common and often misleading social belief about the safety of these drugs. However, a limited selectivity of the first generation NSAIDs towards the COX-2 results in a risk of serious side effects [69]. Adverse effects of zileuton, a 5-LOX inhibitor, were reported as well [70]. Hence, natural products with anti-inflammatory potential have gained the interest of the scientific community [71,72].

According to the data from the literature, both the single phytochemicals that are found in rhubarb species and extracts originating from these plants can modulate inflammatory response at different stages of its development. Following a medicinal approach targeting the arachidonic acid metabolism, numerous natural compounds and extracts of diverse plant origins have been tested in terms of inhibitory action towards COX-2 [73], 5-LOX [74], and even dual COX-LOX inhibitory activity [75]. The COX-2 and/or 5-LOX-inhibitory effects were also found in cellular experimental systems for compounds that are present in rhubarbs, such as stilbenoids [76] and anthraquinones [77], though data are inconsistent. In vitro, the COX-2 inhibitory efficiency of stilbenes reported by different research groups was ranging in micromolar concentrations. For instance, the IC_50_ for rhapontigenin was established to be 36.1 μM, and for resveratrol it attained 3.4 μM [78]. In other work, an in vitro screening of rhaponticin and rhapontigenin inhibitory effects on COX-2 and 5-LOX demonstrated slight or moderate effects of these stilbenoids (at conc. of 1–50 μg/mL) on both enzymes. In contrast, extracts from the roots of *R. rhaponticum* and *R. rhabarbarum* inhibited the COX-2 enzyme with IC_50_ values of 19.16 μg/mL and 19.44 μg/mL, respectively, but their inhibitory effects on 5-LOX were marginal [79].

The use of cell lines enabled the obtaining of some more detailed data on the molecular mechanisms of the anti-inflammatory action of rhubarb. In the cell, one of the most important mediators of inflammatory response development is the NF-κB (nuclear factor kappa-light-chain-enhancer of activated B cells) transcription factor. It regulates the expression of over 400 genes, including those involved in innate and adaptive immune functions and the development of inflammation. Activation of the NF-κB is also a key mechanism of amplifying the inflammatory response and the inflammasome assembly [80,81]. Several phytochemicals of diverse carbon backbones and plant origins (incl. rhubarb) have been found to inhibit the NF-κB-related pathways involved in the pro-inflammatory response [82]. Rhein, emodin, and aloe-emodin have been found to exert at least dual-target (NF-κB, iNOS) inhibition of inflammatory response [83]. Various biological actions of emodin, including anti-inflammatory actions, have been reviewed elsewhere [84]. More recent studies on rhubarb free anthraquinones (RhA) (rhein/diacerein, emodin, aloe-emodin, and 1,8-dihydroxyanthraquinone) conducted in the context of a treatment of the NAFLD have also revealed their ability to ameliorate the inflammatory response by inhibiting the nucleotide-binding oligomerization domain-like receptor protein 3 (NLRP3) inflammasome [85]. In other work, inhibition of the NLRP3/caspase-1/gasermin D (GSDMD) pyroptotic pathway was indicated as a key mechanism of protective action of free total rhubarb (*R. palmatum*, *R. officinale*, and *R. tanguticum*) anthraquinones on the intestinal mucosal barrier of severe acute pancreatitis in rats [86]. Another work has shown stilbenes isolated from *R. rhabarbarum* able to activate the nuclear factor (erythroid-derived 2)-like 2 (also known as the Nrf2 transcription factor) and its downstream antioxidant pathways, including the heme oxygenase-1 (HO-1), an important anti-inflammatory, antioxidant, and cytoprotective enzyme. In mice, treatment with deoxyrhapontigenin (2.5 and 10 mg/kg of body weight (b.w.)) significantly reduced lung inflammation induced by lipopolysaccharide (LPS). This anti-inflammatory effect was displayed through the upregulation of HO-1 and Nrf2, along with simultaneous inhibition of expression of p65, a critical component of the NF-κB [87].

The ability of rhaponticin, rhapontigenin, and root and stalk extracts from *R. rhabarbarum* and *R. rhaponticum* to inhibit the inflammasome formation has been demonstrated in THP1-ASC-GFP reporter cells as well [88]. The PI3K-Akt-mediated NF-κB pathway was also found to be a key molecular mechanism of anti-inflammatory action of *R. palmatum* stem extract [44]. Furthermore, data reviewed by Liu and co-authors [89] suggest that rhubarb-derived extracts and compounds may modulate macrophage polarization during the atherosclerosis-associated inflammation, which subsequently may result in decreased count of M1 macrophages and reduced formation of foam cells. Macrophages can polarize into two main phenotypes: either the pro-inflammatory M1 or the anti-inflammatory M2 phenotype. M2 macrophages are involved in the suppression of inflammation and promote tissue repair, and their formation is induced by anti-inflammatory and immunomodulatory cytokines, including IL-4 and IL-13. In contrast, the formation of M1 macrophages is stimulated by pro-inflammatory factors (e.g., TNF-α, IL-1β, and IL-6) [90] and leads to the augmentation of inflammatory response and progression of atherosclerotic lesions. Due to their pro-inflammatory and pro-atherogenic activity, M1 macrophages strongly contribute to the pathogenesis of atherosclerosis. Therefore, therapeutic strategies (including those employing substances of plant origin) aimed at modulating macrophage polarization to reduce the M1 macrophage pool are considered promising for the prevention of the progression of atherosclerosis [89].

Although studies on isolated compounds provide valuable mechanistic insights into their molecular modes of action, their findings can rarely be directly translated into the biological activities of the extracts [91]. In everyday life, rhubarb is usually found in diets and treatments as a complex mixture of compounds, and its biological effects may result from the synergistic action of various constituents. Therefore, results of studies employing standardized rhubarb preparations with a well-characterized phytochemical profile are crucial for assessing the anti-inflammatory effectiveness of these plants. The rhubarb extracts were found to suppress inflammatory response by triggering receptors, signal transduction pathways, transcription factors, pro-inflammatory enzymes, and secretory processes (data summarized in Table 3).

### 5.2. Reduction in Oxidative Stress

Inflammation and oxidative stress form a tightly interconnected network of biological processes, strongly involved in a number of civilization diseases, including cardiovascular disorders. Once activated, inflammatory cells release reactive oxygen and nitrogen species (ROS/RNS) at the site of inflammation, increasing their levels locally, which can result in oxidative stress. In turn, ROS/RNS are able to initiate or amplify signaling pathways, leading to the enhancement of pro-inflammatory gene expression and strengthening the inflammatory response of the cell [94]. Although antioxidant activity is one of the most extensively studied properties of natural compounds and plant products [95], this type of rhubarb biological activity has only been partly recognized. In addition, the interpretation of existing data is significantly hindered by several factors, such as a limited number of studies, variability in laboratory protocols, and inconsistent data presentation. To date, available evidence of the radical-scavenging activity and antioxidant properties of rhubarb extracts or individual compounds isolated from these plants mostly derives from in vitro assays, including 2,2′-diphenyl-1-picrylhydrazyl (DPPH^•^) and 2,2′-azino-bis(3-ethylbenzothiazoline-6-sulfonic acid (ABTS^•+^) radical-scavenging tests and experiments employing cell lines (Table 4).

The literature indicates that rhubarb-derived extracts and compounds may also reduce damage caused by physiologically relevant oxidants and nitrative agents. The petiole and root extracts from *R. rhabarbarum* and *R. rhaponticum* were found to reduce both the nitrative and oxidative damage of human plasma proteins and lipids, induced by peroxynitrite in vitro. Additionally, they restored or even enhanced the antioxidant capacity of plasma and decreased the peroxynitrite-mediated damage to the fibrinogen structure. Fibrinogen is a key protein of the blood coagulation cascade, and therefore, maintaining its functionality is critical for the hemostasis. Both rhubarb extracts and the two stilbenes (i.e., rhaponticin and rhapontigenin) significantly reduced damage to tyrosine and tryptophan residues in fibrinogen and decreased the extent of peroxynitrite-induced formation of its aggregates [88]. Antioxidants present in rhubarb may counteract oxidative stress by modulation or enhancement of various mechanisms of the cellular antioxidant defense system. For instance, Kalpana and co-authors [96] reported both the signalingmodulatory activity and the ROS (i.e., nitric oxide, superoxide anion, and hydroxyl radical)-scavenging ability of methanol extract from *R. rhabarbarum* (syn. *Rheum undulatum*). Given its demonstrated antioxidant, antibacterial, and cytotoxic properties, the authors suggested that *R. rhabarbarum* extract could serve as a potential additive or preservative in food products, as well as an antibacterial and antioxidant agent in various applications.

**Table 4:** Brief summary of main data on the radical-scavenging and antioxidant activities of rhubarb-derived compounds and extracts.

Rhubarb Species	Plant organ and Examined Substances	Assay Type	Results	Results for Reference Antioxidants	Reference
*R. emodi*	n-hexane, n-butanol, ethyl acetate, dichloromethane, and water rhizome extracts	ABTS^•+^ and DPPH^•^ assays	EC_50_ ranged from 21.52 to 2448.79 μg/mL and 90.25 to 1718.05 μg/mL, for DPPH^•^ and ABTS^•+^, respectively	ascorbic acid EC_50_ = 70.33 and 111.06 μg/mL, for DPPH^•^ and ABTS^•+^, respectively	[97]
*R. palmatum*	methanol extract from stems	DPPH^•^ and O_2_^•^^−^-scavenging tests; ROS scavenging in RAW 264.7 cells	EC_50_ = 290 μg/mL and 480 μg/mL, respectively; ↓ NO production in RAW 264.7 cells; increase in SOD activity in RAW 264.7 cells	EC_50_ = 23.00 μg/mL for ascorbic acid in DPPH^•^ tests	[44]
*R. rhaponticum*	root/rhizome infusion	DPPH^•^ scavenging test	AOX (anti-oxidative efficiency) < 87%	AOX for ascorbic acid (1 mg/mL) was of 77%	[98]
	stalk infusion and ethanolic extract	DPPH^•^ scavenging test	AOX of 48 and 98%, respectively		[98]
*R. rhabarbarum*	the rhizome-derived rhapontigenin and rhaponticin	DPPH^•^ scavenging test and antioxidant action in V79-4 cells	higher efficiency of rhapontigenin; enhancement of cell antioxidant activity; modulation of signaling pathways		[99]
	stalk extract	DPPH^•^ scavenging	DPPH^•^-scavenging efficiencies of the ethyl acetate and methanol extracts (5 µg/mL): 94.12% and 96%, respectively		[100]
	methanol extracts from petioles	ABTS^•^^+^ and DPPH^•^ scavenging assays; FRAP	in the ABTS^•+^, DPPH^•^, and FRAP assays, the “*Red Malinowy*” variety attained 18.26, approx. 5.9 and 10 mmol/Trolox equivalents/100 g of dry mass (d.m.), respectively		[56]
	n-hexane, ethyl acetate, ethanol, acetone, and water rhizome fractions	ABTS^•+^ and DPPH^•^ scavenging assays	EC_50_ ranged from 5.67 to 93.23 μg/mL and from 41.37 to 1800.87 μg/mL, for ABTS^•+^ and DPPH^•^, respectively; the ethyl acetate extract was the most efficient one	gallic acid EC_50_ = 3.16 and 1.25 μg/mL, Trolox EC_50_ = 13.53 and 6.28 for ABTS^•+^, and DPPH^•^, respectively	[101]
	7 fractions (water, 20, 40, 60, 80, and 100% methanol or acetone) isolated from petioles	experimental model of cod liver oil	the highest antioxidant activity found for 100% methanol fraction, i.e., 72.6 and 92.8% at concentrations of 20 and 100 μg/mL, respectively	antioxidant efficiency of BHT: 92.2% at 100 μg/mL; α-tocopherol: 84.8% at 100 μg/mL	[102]
	the rhizome-derived rhaponticin, rhapontigenin, isorhaponticin, deoxyrhaponticin, deoxyrhapontigenin, and resveratrol	ROS scavenging in RAW 264.7 cells	deoxyrhapontigenin IC_50_ = 32.83 μM and 28.22 μM, for ROS and peroxynitrite generation, respectively; induction of HO-1 and activation of Nrf2 via the PI3K/Akt pathway	resveratrol IC_50_ = 49.07 μM for ROS and 37.82 μM for peroxynitrite, respectively	[87]
	the rhizome-derived piceatannol, resveratrol, rhapontigenin, deoxyrhapontigenin, pterostilbene, (E)-3,5,4′-trimethoxystilbene, and trans-stilbene	HepG2 cell line exposed to arachidonic acid + iron-induced oxidative stress	reduction in the oxidative stress-induced mitochondrial dysfunction through AMPK pathway		[103]
*R. ribes* L.	chloroform and methanol root and stem extracts	DPPH^•^ and O_2_^•^^−^ scavenging tests; Fe^3+^ and Cu^2+^-reducing assays; β-carotene bleaching; ion chelation	in most tests, extracts displayed considerable efficiency when compared to reference antioxidants	ascorbic acid, α-tocopherol, BHA, BHT, quercetin	[104]
	stem water, ethanol and methanol extracts	ABTS^•+^, DPPH^•^ and ^•^OH scavenging assays	ABTS^•+^ scavenging efficiency: 99.27, 99.91, and 99.88%; DPPH^•^ scavenging efficiency: 83.11, 81.42, and 83.26%; ^•^OH scavenging efficiency: 93.49, 94.21, and 95.86%, for stem water, ethanol and methanol extract, respectively	BHA scavenging efficiency: 95.32, 80.49, and 93.78%, for ABTS^•+^, DPPH^•^, and ^•^OH, respectively	[105]
*R. officinale*	methanol extract from petioles	DPPH^•^, ^•^OH, O_2_^•^^−^ scavenging assays; antioxidant activity in RAW 264.7 cells	EC_50_ in DPPH^•^-scavenging = 205.13 μg/mL ↓ lipid peroxidation protection of SOD, CAT activity	ascorbic acid EC_50_ in DPPH^•^-scavenging = 19.33 μg/mL	[43]
*R. tanguticum*	methanol extract from petioles	DPPH^•^, ^•^OH, and O_2_^•^^−^ scavenging assays; antioxidant activity in RAW 264.7 cells	EC_50_ in DPPH^•^-scavenging = 497.03 μg/mL ↓ lipid peroxidation protection of SOD, CAT activity		[43]
*R. telianum* İlçim	ethanol extracts from leaves and seeds	DPPH^•^ scavenging test Fe^3+^, Cu^2+^-reducing tests, FRAP assay	EC_50_ = 20.79 and 5.67 μg/mL, respectively, seed extract was the most effective in all reducing tests	EC_50_ = 16.00, 12.99, 9.63, and 13.92 μg/mL, for BHA, BHT, Trolox, and ascorbic acid respectively	[106]
*R. turkestanicum* Janischew	ethanol root extracts	pheochromocytoma (PC12) and neuroblastoma (N2a) cells	↓ lipid peroxidation ↓ ROS generation ↓ apoptosis		[107]

↓—decrease; ^•^—radical.

### 5.3. Anti-Obesity and Cardioprotective Effects

The cardioprotective properties of natural and synthetic substances involve diverse biological and/or pharmacological activities resulting in a reduction in the risk of developing cardiovascular diseases. Given the well-documented association of obesity and hyperlipidemia with the occurrence of cardiovascular events [108], prevention and treatment of hypercholesterolemia, obesity, and other symptoms included in the metabolic syndrome criteria constitute crucial dietary and medical approaches to reduce cardiometabolic disease-associated mortality. Another factor related to the development of cardiovascular diseases is obesity-associated inflammation. Systemic and adipose tissue inflammation is tightly related to obesity-associated cardiovascular diseases and type 2 diabetes mellitus [109].

Owing to a significant amount of natural fiber content, rhubarb petioles were originally investigated in the context of their cholesterol-lowering efficiency. The first studies on this issue were performed as early as the 1990s and have continued for over two decades. Both examinations on animal models of hypercholesterolemia and studies on human subjects demonstrated the ability of rhubarb fiber to decrease the plasma cholesterol level. Moreover, the fiber had beneficial effects on the liver and gallbladder functions [110]. However, recent decades have indicated that the beneficial effects of rhubarb-derived substances on human health (including their lipid-lowering properties) have a more complex molecular and physiological basis. Furthermore, the health-promoting potential of low-molecular phytochemicals has been highlighted, along with growing evidence of the synergistic interactions among various classes of plant metabolites. Hence, the hypothesis that the hypolipidemic properties of rhubarb are mainly due to its fiber content has been reconsidered. For instance, in vivo anti-hyperlipidemic effects have been found for stilbene compounds. Resveratrol has been reported to improve blood plasma lipid profile [111,112,113], stimulate thermogenesis [114], and reduce insulin resistance [115]. Beneficial effects on liver physiology and lipid-improving properties were also found for rhaponticin and rhapontigenin isolated from the roots of *R. rhabarbarum* [116,117]. Evidence from the literature indicates that the hypolipidemic and anti-obesity effects of rhubarb-derived extracts and individual compounds are a result of acting on diverse molecular levels. The impact of rhubarb-derived substances on the adipose tissue may be a result of a reduction in cholesterol biosynthesis [118], the inhibition of key enzymes responsible for lipid absorption [119], the regulation of adipogenesis [120,121], and the modulation of mature adipocytes’ metabolism [122,123]. Aloe-emodin, rhein, and emodin were found to be the main cholesterol-regulating components of RhA in an animal study. Examinations of molecular mechanisms of cholesterol-lowering action of RhA revealed their modulatory effect on the expression of key regulators of cholesterol synthesis and metabolism, i.e., the sterol-regulatory element binding protein 2 (SREBP2), 3-hydroxy-3-methyl glutaryl coenzyme A reductase (HMGCR), and squalene monooxygenase (SQLE) [124].

The anti-inflammatory effects on the white adipose tissue have been revealed for some metabolites synthesized by different rhubarb species [125]. Rhubarb supplementation for mice was found to prevent diet-induced obesity, diabetes, visceral adiposity, adipose tissue inflammation, and accumulation of liver triglyceride, as well as to promote the growth of *Akkermansia muciniphila*, a commensal bacterium exerting health-promoting properties [126]. Moreover, the beneficial effects of rhubarb supplementation on metabolic disorders induced by high-fat and high-sugar diet in mice were enhanced when combined with inulin (i.e., 0.3% rhubarb in the diet was combined with 20% inulin provided in the drinking water) [127].

Some of the outcomes of in vitro and animal studies on lipid-lowering and anti-obesity properties of rhubarb have already been confirmed in humans. Treatment with *R. officinale* hot water extract (50 mg/kg b.w.) effectively decreased the serum total cholesterol and the LDL cholesterol in patients with diagnosed atherosclerosis (a randomized, double-blind and placebo-controlled trial) [128]. Recently published results of a double-blind, randomized human clinical trial involving 52 healthy participants have indicated that consumption of green tea with rhubarb (*R. rhabarbarum*) root for 21 days significantly lowered total cholesterol, LDL-cholesterol, and non-HDL cholesterol levels. Moreover, this combined *Camellia sinensis* L. and *R. rhabarbarum* tea formulation demonstrated beneficial effects in maintaining gut eubiosis and did not induce any observable adverse effects [129]. On the other hand, the *R. emodi* stem extract (a total amount of 90 extract capsules: 3 capsules/day, and 400 mg of the extract/capsule) did not influence the body weight of patients with type 2 diabetes mellitus; however, the extract had some beneficial effect—a decrease in systolic and diastolic blood pressure [130].

Furthermore, a very recent review on rhubarb-derived (particularly *R. palmatum*) substances used in TCM to treat atherosclerosis has focused on pharmacology and possible mechanisms of anti-atherosclerotic actions of the main rhubarb bioactive compounds, i.e., various anthraquinones, stilbenes, phenolic acids, and polysaccharides. It was primarily devoted to presenting the great potential of rhubarb-derived compounds in their possible application for the treatment of atherosclerosis; however, it also highlighted that problems regarding the toxicity profile of rhubarb still remain unresolved in light of the existing experimental data and require further interdisciplinary studies aiming at final approval for the clinical use of rhubarb [131].

### 5.4. Anti-Inflammatory Effects of Rhubarbs in the Digestive System

In both traditional and modern phytomedicine, roots of *R. officinale* and *R. palmatum* are recognized for their laxative effects, primarily due to a high anthraquinone content (up to 5% of d.w.) [132,133] and the presence of compounds stimulating intestinal motility, such as anthrones and dianthrones (e.g., rheinosides A-D, palmidins A-C, rheidins A-C, and sennosides A-F) [134]. The intestinal metabolism of natural substances is critical for their bioavailability and, in consequence, their biological effects [135,136]. In the case of rhubarb, this fact is particularly important. Due to laxative effect and the risk of irritating the intestinal mucosa, for many years, the above-mentioned rhubarb species have not been considered a source of intestinal-protective substances. Also, the safety of administration of rhubarb-derived preparations, especially those based on the roots of anthraquinone-rich species (e.g., *R. palmatum*, *R. officinale*, or *R. tanguticum*), has been discussed, especially in the context of possible abuse and/or adverse effects [137,138]. Due to significant differences in immune response and liver functions found in normal and diseased rats, it has been suggested/emphasized that taking rhubarb preparations could be associated with a risk of hepatotoxicity in patients suffering from certain diseases [139].

On the other hand, a growing body of evidence indicates much more complex interactions of rhubarb-derived compounds in the digestive system, which depend not only on the type of preparation used, but also on the dose administered. For instance, *R. emodi* (belonging to the *Palmata* section), which is traditionally recommended for purgative therapy in Asian countries, may evoke diverse physiological effects, dependent on the dose. While at larger doses *R. emodi* acts as a natural laxative, in small doses, this plant is used to treat dysentery and diarrhea [140]. Although the presence of anthraquinones in rhubarb extracts is still believed to be a significant obstacle to their oral use and application to treat diverse diseases of the digestive system, various successful attempts in animal studies on the therapeutic use of either preparations with native levels of anthraquinones or those with reduced content of these compounds have been described. Administration (400 mg/kg b.w. daily) of the fermented rhubarb (*R. rhabarbarum*) preparation with a reduced anthraquinone content was found to significantly reduce the metabolic dysfunction-associated steatotic liver disease in mice. The beneficial effects of fermented rhubarb were due to its ability to modulate the composition of the gut microbiome and to activate the insulin signaling pathway in the liver [141]. Treatment with *R. officinale* decoction was found to relieve constipation by stimulating the secretion of mucus in the large intestine. Although it has been established that the molecular mechanisms of this action involve the activation of mast cells and the submucosal nervous system, the exact proportions of these two mechanisms have not been fully estimated [142]. In another in vivo study, anti-constipation therapy based on rhubarb root extract ameliorated its occurrence in animals and suppressed constipation-associated inflammation [143]. Anti-inflammatory effects within the gastrointestinal system and the ability to ameliorate ulcerative colitis (UC) have also been reported for *R. officinale* leaf juice. These effects were attributed to the inhibition of the NF-κB/NLRP3 signaling pathway, as evidenced by reduced levels of p-p65, p-IκBα, NLRP3, and ASC. The inhibition of key pro-inflammatory pathways resulted in the alleviation of inflammatory response as well as the reduction in oxidative stress and intestinal injury in mice [144]. Rhubarb-including therapies to treat UC in Chinese medicine with a purgative effect have been shown to therapeutically influence UC by regulation of microflora in the intestine, improving intestinal microcirculation, and exerting anti-inflammatory action. To assess the efficacy and safety of UC rhubarb-based (*R. palmatum*) therapy, a meta-analysis including randomized clinical trials has been performed. Results from 30 studies have shown that therapy of UC by applying rhubarb preparations could improve clinical outcomes and reduce the recurrence rate. Interestingly, rhubarb-containing preparations in combination with 5-aminosalicylic acid (5-ASA) or sulfasalazine (SASP) were found to be more effective than 5-ASA or SASP used alone. Moreover, a meta-analysis did not reveal significant side effects displayed by rhubarb-based UC therapy, which could be recommended to be applied within 1–13 weeks or 3 months and could be administered orally or by enema [145].

*R. tanguticum* and *R. officinale* decoctions have been demonstrated to exert anti-cholestatic effect [146] and attenuate the severity of acute necrotizing pancreatitis [147], respectively. In an animal model of alcoholism, a treatment with cooked rhubarb (3 g/kg b.w./day) alleviated inflammation and other changes in pancreatic tissue induced by chronic alcohol exposure. Interestingly, this study demonstrated the ability of rhubarb to influence the epigenome, including modulation of pro-inflammatory and anti-inflammatory mediators by regulating DNA methylation and IL-1α and IL-10 protein expression [148]. The protective effect of rhubarb on liver functions in animals exposed to acute alcohol intake was also reported. Diet supplemented with 0.3% rhubarb extract for 17 days prevented hepatic inflammation induced by acute alcohol intake by downregulating mediators of inflammatory response and oxidative stress, including the Toll-like receptor 4-dependent signaling. The rhubarb supplementation also resulted in regulation of the gut microbiota profile and improvement in intestinal homeostasis [149].

Importantly, the anti-inflammatory effects of rhubarb have also been found in a randomized controlled trial. The rhubarb preparation combined with early enteral nutrition was administered to patients with severe acute pancreatitis (SAP), and the therapy resulted in a significant improvement of gastrointestinal function, reduction in systemic inflammation parameters and disease severity, and the mitigation of the disease-related damage to liver and kidney functions [150]. To further verify the clinical efficiency of a rhubarb preparation in combination with enteral nutrition, a meta-analysis was also performed. The analysis involved results of eleven randomized controlled trials and confirmed that the combination of rhubarb with early enteral nutrition may reduce both systemic inflammation and disease severity, as well as enhance the effectiveness of enteral nutrition in SAP patients [151].

The role of the microbiome, including participation of intestinal microbiota and the gut–brain axis in immunity, nutrient synthesis, and modulation of many other processes at the systemic level, attracts much research attention [152,153]. Furthermore, a strong relationship between the occurrence of metabolic disorders, inflammation, and gut microbiota composition has been well established [154]. Following these research trends, different rhubarb species have also been investigated as potential modulators of the human microbiome, especially (but not only) in the context of gut microbiota-associated disorders. Modulation of intestinal epithelial microbiota has been listed as one of the possible mechanisms by which rhubarb may promote intestinal mucosal innate immune homeostasis [155]. A stewed *R. palmatum* decoction was found to modulate a restoration of destroyed gut microbiota and ameliorate chronic renal failure in mice [156] as well.

The latest animal studies have indicated that rhubarb extracts may have an anti-inflammatory effect in the digestive system [157], improve the gut microbiota dysbiosis [158], and even possess anti-ischemic potential [159,160]. The administration of raw rhubarb (*R. officinale*) preparation to rats modulated the bile acid metabolism and significantly attenuated gut microbiota dysbiosis evoked by ischemic stroke. Experiments involving the pseudo-germ-free animals have indicated that the anti-stroke effect of *R. officinale* may be dependent on its effects on intestinal microbiota [161].

### 5.5. Rhubarb-Derived Polysaccharides as Immunomodulatory and Anti-Inflammatory Agents

In addition to being rich in dietary fiber and low-molecular-weight phytochemicals, rhubarb is also a significant source of polysaccharides—natural compounds known for their beneficial effects on both intestinal and systemic health [131]. Polysaccharides can affect human health via microbiota-dependent and microbiota-independent pathways [162], resulting in immunomodulatory and anti-inflammatory effects, which are considered particularly important for patients suffering from inflammatory bowel disease as well as other conditions associated with chronic inflammation. The efficiency of rhubarb polysaccharides in the management of various disorders and their ability to modulate diverse pro-inflammatory mechanisms, including the Notch and NF-κB signaling pathways, have been confirmed as well. Administration of *R. tanguticum*-derived polysaccharides (100–300 mg/kg b.w.) has been found to ameliorate UC in mice. Analysis of inflammatory markers in treated animals revealed a significant reduction in pro-inflammatory cytokines (IL-6, IL-1β, and TNF-α) and oxidative stress biomarkers [163]. In another study, *R. tanguticum* polysaccharides (500 mg/kg b.w.) ameliorated radiation-induced enteritis via activation of Nrf2/HO-1 in rats [164].

The anti-inflammatory action of rhubarb polysaccharides may be enhanced through combination with other plant-derived preparations, potentially eliciting synergistic effects. For instance, promising results in colitis management in animals were obtained for the combination of polysaccharides from *R. palmatum* with *Coptis chinensis* Franch. [165] and for *R. officinale* polysaccharides administered together with *Semen Crotonis Pulveratum* (a traditional preparation from croton plant seeds) [166]. Another way of enhancing the anti-inflammatory efficiency of rhubarb polysaccharides may be by incorporating them into targeted drug delivery nanosystems. In an animal model, treatment with nanoparticles composed of co-assembled *R. palmatum* polysaccharides and berberine (extracted from *C. chinensis*), at the dose of 225 mg/kg b.w., was found to ameliorate UC by regulation of the intestinal flora [167].

Moreover, accumulating evidence indicates that targeting gut microbiota with polysaccharides is a very promising medical approach in the treatment of metabolic diseases, especially those related to a high-fat diet [168,169]. Recent animal study on the efficiency of rhubarb polysaccharides on NAFLD has demonstrated that a dietary intervention based on these substances (at daily doses of 270 or 540 mg/kg b.w.) resulted in a multilevel improvement of liver functions. The treatment inhibited lipid accumulation in the liver tissue, reduced inflammatory damage to hepatocytes, modulated the bile acid and fatty acid metabolism, and maintained the functions of the gut barrier. At a molecular level, such dietary supplementation exerted metabolic restorative effects by improving bile acid transporter activity, inhibiting fatty acid synthesis and transport, and stimulating hepatic β-oxidation of fatty acids [170].

## 6. Future Prospects and Challenges

Although the existing literature provides strong evidence for anti-inflammatory properties of different rhubarb species, several research and application challenges remain. Firstly, a reliable assessment of the anti-inflammatory efficiency of rhubarb-based foods at the physiological level is still necessary. Many works on anti-inflammatory, antioxidant, and other biological effects of rhubarb are preliminary and require more advanced approaches, including in vivo studies. While the roots of *R. officinale* and *R. palmatum* are widely recognized as the primary source of plant material commonly known as the *Rhei radix*, limited data exist on the bioactive properties of their petioles. Also, the nutritional value and biological properties of other species from the *Palmata* section are insufficiently determined. Furthermore, the evidence in the literature suggests that despite their phylogenetic proximity, species from the *Rhapontica* section may influence distinct molecular pathways and exhibit significant differences in anti-inflammatory efficacy [79,88].

Secondly, there is a risk of toxic action, especially when the isolated substances or extracts are used. The risk of toxicity of rhubarb-derived substances (with a special emphasis on concoctions with a concentrated pool of anthraquinones) should be established and eliminated. So far, the molecular mechanisms underlying the rhubarb-induced toxicity remain unclear, and a need for further evaluation of emodin and rhein has been discussed in the literature [171]. Another important issue is the presence of oxalates in rhubarb-derived foods and the possibility of reducing them by using various food-processing techniques. Different modes of food processing, such as boiling, fermentation, and cooking with calcium sources, have been proposed in the literature to reduce the oxalate level in food. In addition, some works recommend consumption of calcium supplements, probiotics, and dairy foods [172]. However, the usefulness of these methods to reduce the intake of rhubarb-derived oxalates has been only partly recognized.

Thirdly, the optimization of the food formula. Developing food product formulations that maximize health benefits remains a challenge. Preliminary evidence suggests that rhubarb can be effectively combined with various fruits and vegetables to provide novel food products. Furthermore, such formulations may be based on by-products, such as pomace, supporting a “zero-waste” approach. However, the issue of preserving sensory attributes when producing new foods is also of key importance. Thus, comprehensive studies on formulation strategies, synergistic interactions, and processing conditions are needed.

## 7. Conclusions

In summary, the literature shows that rhubarb exhibits pleiotropic biological activity at both molecular and organ levels. In light of the results of scientific investigations, different rhubarb species traditionally used in Chinese medicine, as well as in the ethnomedicine of other cultures to treat various disorders, were shown to possess a significant potential in the alleviation of inflammation-related diseases. These effects may be displayed through the suppression of genes for pro-inflammatory cytokines, enzymes, and other mediators of inflammation, direct inhibition of pro-inflammatory enzymes, modulation of immune responses, and regulation of metabolic pathways. In light of the literature data, antioxidant properties of rhubarb extracts or individual compounds isolated from these plants may additionally enhance health-promoting properties of rhubarb by mitigating the inflammation-associated oxidative stress. Both the extracts and isolated compounds exert considerable radical-scavenging and antioxidant properties in vitro. Nevertheless, the antioxidant activity of rhubarb extracts and foods remains insufficiently characterized, particularly in in vivo studies. Therefore, further studies are needed to evaluate the physiological significance of the antioxidant activity of rhubarb-based foods.

Notably, beneficial effects have been observed for both rhubarb low-molecular-weight phytochemicals and natural polymers such as dietary fiber and polysaccharides. Rhubarb preparations have good chemical composition, e.g., high flavonoid and polysaccharide content, and therefore, they can be proposed as ingredients of healthy food or dietary supplements. However, due to a variability in laboratory protocols and inconsistent data presentation, more studies on standardized rhubarb extracts are needed to facilitate their possible use in a diet alleviating both inflammation itself and inflammation-related diseases.

## Figures and Tables

**Table 1:** Pro-inflammatory factors and main mechanisms of plant-derived foods to prevent or ameliorate inflammation induced by various factors (compilation of data from the literature [8,9,10,11]).

Type of Pro-Inflammatory Factors	Examples	Activities That May be Particularly Relevant for Anti-Inflammatory Effects of Food Components
physical and chemical factors, including environmental pollutants	ultraviolet and ionizing radiation, xenobiotics (including pesticides), heavy metals, endocrine disruptors, allergens	different mechanisms of antioxidant action, incl. scavenging of reactive oxygen and nitrogen species, stimulation of the antioxidant enzyme-mediated protection, transition, and heavy metal ion chelation; suppression of pro-inflammatory response at the molecular and cellular level, toxin-binding ability of dietary fiber
pathogens and other biological factors	infections, microbiome dysbiosis	antibacterial, antiviral, or antifungal action; immunomodulatory activity; the physiological microbiome-supporting properties
lifestyle-related	sedentary lifestyle, alcohol, smoking, unbalanced diet, consumption of highly processed and high-calorie foods, chronic stress	antioxidant properties; detoxifying effects; metabolism-modulatory properties and anti-obesity action; cardioprotective and hepatoprotective actions
diseases	obesity, insulin resistance, diabetes, and other chronic metabolic disorders, autoimmune diseases	antioxidant properties; suppression of pro-inflammatory response at the molecular and cellular level; metabolism-modulatory properties and anti-obesity action; cardioprotective action

**Table 2:** Exemplary data on ethnomedicinal uses of different rhubarb species and various parts of these plants.

Species	Plant Organ and/or Type of Preparation	Ethnomedicinal Recommendations	References
*R. acuminatum* Hook.f. & Thomson	leaves and petioles	diarrhea, headaches, constipation; traditional food: as a vegetable or used to prepare pickles	[15]
*R. australe* D.Don.	root powder or roots in different formulations	cough and rhinitis, hemoptysis, as a laxative, tonic, and diuretic to improve wound healing	[16]
	pounded fruit	herpes infection	[17]
*R. emodi* Wall.	roots paste mixed with turmeric powder and mustard oil	rheumatism, muscular pain	[18]
	root decoction mixed with oil	burns	[19]
*R. maximowiczii* Losinsk.	roots-based preparations	intestinal disorders, constipation, or diarrhea	[20]
	crushed leaves and petioles	wounds	[20]
*R. officinale* Baill.	roots in different formulations	gastrointestinal disorders, blood purification, detoxification, fever, removing blood stasis, promoting menstruation	[21]
		chronic renal failure	[22]
		cancer	[23]
*R. palmatum* L.	root-based preparations	constipation, gastritis, hepatitis, gastric ulcer, enteritis, diabetes, inflammation, atherosclerosis, cancer	[24]
*R. rhabarbarum* L.	root-based preparations	liver, spleen, and stomach dysfunctions blood purification, bleeding, fever, injuries, and trauma	[25]
	roots in different formulations	a natural anti-inflammatory agent for appendicitis, cholecystitis, and rheumatoid arthritis therapy	[26]
*R. rhaponticum* L.	roots in wine, beer, or mead	gastrointestinal pain, gastritis, liver and spleen disorders, heartache and pain in pericardium, pulmonary system dysfunctions, reproductive system disorders, uterine, and breast pain	[25]
	roots boiled in red wine and sweetened with honey	to improve voice	[27]
	sugar syrup	fever	[27]
	a brandy-based balsam	heart problems or stomachache	[27]
	underground parts	ingredient of the *Mesir* paste—a traditional Turkish folk food and remedy, used to treat infectious diseases and to stimulate the immune system	[28]
*R. tanguticum* Maxim. ex Balf.	dried root powder	purgative	[29]
*R. tibeticum* Maxim. ex Hook.f.	roots	expectorant, injuries, ulcers, skin ailments	[30]
	boiled leaves	laxative	[30]

**Table 3:** Anti-inflammatory effects of rhubarb extracts found in different cellular experimental models.

Rhubarb Species	Type of Extract	Experimental System	Main Findings	References
*R. palmatum*	stem extract	RAW 264.7 cells	↓ NO, IL-1β, IL-6, and TNF-α	[44]
*R. rhaponticum*	root extract	PBMCs	↓ TNF and IL-2 release	[88]
		HUVECs	no changes in COX-2 gene expression ↓ 5-LOX gene expression ↓ monocyte adhesion to endothelial cells	[79]
		THP1-ASC-GFP monocytes	↓ inflammasome activation	[88]
	petiole extract	PBMCs	↓ TNF and IL-2 release	[88]
		HUVECs	↓ COX-2 gene expression ↓ 5-LOX gene expression	[79]
		THP1-ASC-GFP monocytes	↓ inflammasome activation	[88]
*R. rhabarbarum*	root extract	PBMCs	↓ IL-2 release	[88]
		HUVECs	no changes in COX-2 gene expression no changes in 5-LOX gene expression ↓ monocyte adhesion to EC	[79]
	rhizome extract	HUVECs	↓ TNF-induced activation of NF-κB-p65 ↓ expression of adhesion molecules (ICAM-1 and VCAM-1) ↓ the monocyte chemoattractant protein-1 (MCP-1)	[92]
		THP1-ASC-GFP monocytes	↓ inflammasome activation	[88]
		RAW 264.7 macrophages	↓ inflammatory activation, incl. generation of NO	[93]
	petiole extract	PBMCs	↓ TNF and IL-2 release	[88]
		HUVECs	↓ COX-2 gene expression ↓ 5-LOX gene expression	[79]
		THP1-ASC-GFP monocytes	↓ inflammasome activation	[88]
		RAW 264.7 macrophages	↓ inflammatory activation, incl. generation of NO	[93]
	leaf extracts	RAW 264.7 macrophages	↓ inflammatory activation, incl. generation of NO	[93]

↓—decrease.

## Data Availability

No new data were created or analyzed in this study.
